# The Role of Salivary Interleukin-6, Interleukin-8, and Epidermal Growth Factor in Radiotherapy Outcomes in Patients With Head and Neck Cancer: A Review of the Literature

**DOI:** 10.7759/cureus.94350

**Published:** 2025-10-11

**Authors:** Stella Fotiadou, Anastasios Stefanidis, Evgenia Daskalaki

**Affiliations:** 1 Otolaryngology - Head and Neck Surgery, Theagenio Cancer Hospital, Thessaloniki, GRC

**Keywords:** epidermal growth factor, head and neck cancer, interleukin 6, interleukin 8, radiotherapy, salivary cytokines

## Abstract

Head and neck cancer (HNC) comprises a heterogeneous group of tumors and is a significant malignancy worldwide. Radiotherapy is a treatment option for HNC patients and is known to elevate pre-inflammatory cytokines. However, cytokines in saliva are not widely studied in HNC patients after radiotherapy. The aim of this literature review is to examine the association of the most frequently analyzed salivary cytokines pre- and post radiotherapy in HNC patients, with the outcomes of the treatment. An analytic search was conducted for this literature review on PubMed and Embase databases. Specific combinations of keywords were used, including salivary cytokines, interleukin-6 (IL-6), interleukin-8 (IL-8), epidermal growth factor (EGF), head and neck cancer, and radiotherapy. Several studies showed an increase in salivary IL-6 and IL-8 post treatment. It was associated with worse oral mucositis, severe toxicity, and treatment outcome. Salivary EGF was decreased post radiotherapy in most cases, and was correlated with worse oral mucositis. A minimally invasive technique, such as salivary sampling, could serve as a baseline prognostic tool. Salivary cytokines may predict which patients will develop severe toxicity, severe oral mucositis, or allow earlier prognosis in the radiotherapy course and outcome.

## Introduction and background

Head and neck cancer (HNC) comprises a heterogeneous group of tumors, 90% originating from squamous cells in the head and neck. This pathology originates from the following anatomical sites: oropharynx, oral cavity, hypopharynx, larynx, nasopharynx, paranasal sinuses, nasal cavity, salivary glands, and thyroid gland [1]. It is the seventh most frequent malignancy worldwide, with approximately 931,931 patients diagnosed per year [1]. HNC usually affects older adults, especially those over age 50. It is more common in men, and alcohol consumption and tobacco use are the main risk factors [2]. Human papillomavirus (HPV) infection, especially HPV-16, is another factor implicated in the carcinogenesis of tumors arising from the oropharynx [3]. Treatment options for HNC are surgical removal of the tumor, radiotherapy, and chemotherapy [4]. Prognosis depends on the stage at the time of diagnosis. HPV infection has better prognostic outcomes. The five-year survival rate is 70-90% for early stages and 40-60% for advanced stages [1,4].

Radiotherapy enhances the levels of cytokines, which are responsible for inflammation, proliferation, and disease progression in HNC, especially in oral squamous carcinoma patients [5]. Cytokines regulate cell proliferation, enhance the formation of new blood vessels, help cancer cells to invade adjust tissues, and promote cell survival [6]. The most frequently analyzed cytokines in saliva are interleukin-6 (IL-6), interleukin-8 (IL-8), and epidermal growth factor (EGF) [7]. These specific proteins are widely used as prognostic biomarkers. Samples of salivary cytokines are more reliable than plasma, because inflammatory conditions impact on plasma levels of cytokines [7]. Salivary cytokines are usually used for the detection and prognosis of HNC patients [6,7]. However, alterations in saliva samples of patients from pre- to post radiotherapy have not been comprehensively explored due to the damage of salivary glands and subsequent xerostomia with conventional radiation [5]. Intensity-modulated radiation therapy (IMRT) is a technology of high-precision radiotherapy that spares the salivary glands and allows stimulated saliva recovery. As a result, salivary biomarkers can be studied with the use of IMRT [8].

HNC patients have a risk of developing recurrent or second primary tumors, and must be monitored after treatment [1,2]. Salivary IL-6, IL-8, and EGF could provide an easy, accurate, and non-invasive monitoring tool for prognosis [7]. The aim of this review of the literature is to examine the association of salivary cytokines pre- and post radiotherapy in HNC patients, with the outcomes of the treatment.

## Review

Methods

Search Strategy of the Literature

**Figure 1:**
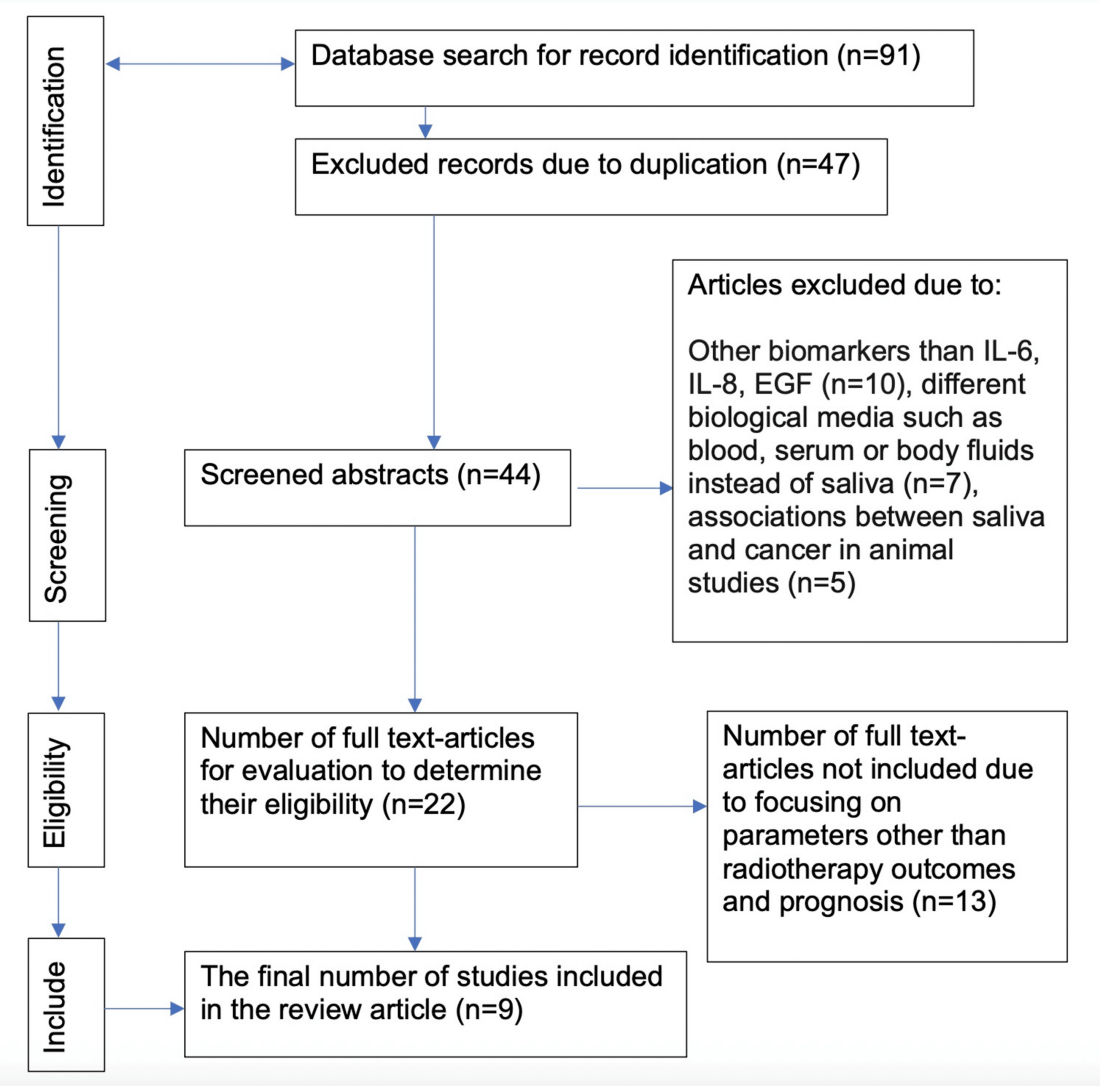
Preferred Reporting Items for Systematic Reviews and Meta-Analyses (PRISMA) flow diagram for literature research strategy. IL-6: interleukin-6; IL-8: interleukin-8; EGF: epidermal growth factor.

An analytic search was conducted for this review of the literature on PubMed and Embase databases following the guidelines of the Preferred Reporting Items for Systematic Reviews and Meta-Analyses (PRISMA) [9]. Specific combinations of keywords were used: (“salivary cytokines” OR “salivary interleukin 6” OR “salivary interleukin 8” OR “salivary epidermal growth factor”) AND (“head and neck cancer”) AND (“radiotherapy”). The findings were limited to articles published in the English literature. The relevant studies have been conducted between 1997 and 2025 (Figure 1). The titles, abstracts, and references of articles were thoroughly studied. Three research team members reviewed each article independently to establish its inclusion, based on the selection criteria. In cases of disagreement in the research team, a consensus method was used. The studies were categorized based on population screened, method of study, type of biomarkers present, and association with oral mucositis.

Selection Criteria

A total of 91 articles pertaining to salivary cytokines in HNC patients treated with radiotherapy were identified. Studies were selected that specifically addressed the role of IL-6, IL-8, and EGF in the saliva of patients after radiotherapy. Included studies used enzyme-linked immunosorbent assay (ELISA), reverse transcription polymerase chain reaction (RT-PCR), and multiplex assay in salivary samples. Studies conducted on blood, serum, and body fluid, or on animal experiments, were excluded. A total of 13 studies that focused on parameters other than radiotherapy outcome and prognosis were also excluded. Nine studies were ultimately selected for analysis. Two studies investigating the role of IL-6, one study investigating IL-8, four studies investigating EGF, and two studies investigating combinations of them.

Results

The studies were conducted between 1997 and 2025, in six different countries: India, Italy, the United States, Spain, Iraq, and Canada [10-18]. The methods assessing the salivary cytokines were RT-PCR, ELISA, multiplex assay, and immunoassays [10-18]. A summary of the main characteristics of selected studies is provided in Table 1.

**Table 1:** Summary of main characteristics of the selected studies. HNC: head and neck cancer; OSCC: oral squamous cell carcinoma; RT-PCR: reverse transcription polymerase chain reaction; ELISA: enzyme-linked immunosorbent assay; IL: interleukin; EGF: epidermal growth factor; GROα: growth-regulated protein alpha; TNF-α: tumor necrosis factor-alpha; VEGF: vascular endothelial growth factor; MCP-1: monocyte chemoattractant protein-1; HNSCC: head and neck squamous cell carcinoma.

Author	Year	Country	Cancer	Cases	Methods	Results
Shree et al. [10]	2024	India	OSCC	60	RT-PCR, ELISA	Post-therapy, IL-6 levels decreased significantly with p < 0.0001, indicating treatment response and further resulted in normalizing to baseline at 6-month follow-up.
Bossi et al. [11]	2016	Italy	HNC	55	Multiplex assay	IL-1β, TNF-α, and IL-6 were elevated during the third week of therapy, related to the development of worse mucositis in the oral cavity.
Russo et al. [12]	2016	USA	HNC	16	Multiplex assay, ELISA	In oropharyngeal HNSCC, secretion of EGF, IL-6, IL-8, TNF-α, IL-1α, IL-β, VEGF, and GROα elevated significantly post-chemoradiotherapy.
Citrin et al. [13]	2012	USA	HNC	11	Multiplex assay	IL-6, IL-8, EGF, IL-4, TNF-α, VEGF, and MCP-1 levels were elevated at the high dose area, in contrast with the low dose area. IL-6 and IL-8 were elevated as the dose of radiation was higher.
Principe et al. [14]	2022	Spain	HNC	30	Immuno-assays	IL-8 could be a predictive biomarker of radiotherapy response. Salivary IL-8 increased statistically significantly in HNC patients, post-treatment, indicating good radiotherapy outcomes.
Alturfi et al. [15]	2025	Iraq	HNC	54	ELISA	Increased EGF expression was reported after radiotherapy and correlated with reduced oral mucositis occurrence. A threshold value for EGF was 502.1 pg/ml, and was related to the occurrence of radiotherapy-induced mucositis in the oral cavity.
Epstein et al. [16]	2000	Canada	HNC	18	ELISA	Higher salivary EGF levels were statistically significantly associated with less severe erythema and ulceration. EGF was substantially reduced in the initial phase of treatment and was consistently reduced during treatment.
Dumbrigue et al. [17]	2000	USA	HNC	13	ELISA	Salivary EGF was decreased, and more severe mucositis was present, with a statistically significant correlation.
Epstein et al. [18]	1997	Canada	HNC	16	ELISA	Salivary EGF was decreased during the course of treatment, and an association was reported with reducing EGF and worsening ulcers in the oral cavity, and elevating the total mucositis score and severity of oral mucositis.

Shree et al., in 2024, showed that IL-6 in patients’ saliva could be a potential prognostic marker in oral squamous cell cancer (OSCC) management [10]. IL-6 expression was measured in 60 cases of both postoperative OSCC patients and controls, in saliva samples, one year post radiotherapy and post chemotherapy. RT-PCR and ELISA were used. Pre-chemoradiotherapy, OSCC patients presented higher expression of IL-6 related to the healthy group. After treatment, the expression of IL-6 reduced subsequently, demonstrating treatment response. IL-6 levels were normalized to baseline at six-month follow-up after therapy [10].

Bossi et al., in 2016, explored the role of salivary cytokines during chemoradiotherapy and their correlation to the severity of oral mucositis in HNC patients [11]. The study consisted of 55 HNC cases, 10 individuals who did not have cancer, and 10 individuals with cancers not located in the head and neck region. Salivary cytokine concentrations were measured with a multiplex assay. Tumor necrosis factor-α (TNF-α), IL-1β, and IL-6 increased during treatment. Particularly, these cytokines were elevated in patients during the third week of therapy. This increase was significantly related to the development of worse mucositis in the oral cavity [11].

In 2016, Russo et al. evaluated the salivary expression of EGF, IL-6, IL-8, TNF-α, IL-1α, IL-β, vascular endothelial growth factor (VEGF), and growth-regulated protein alpha (GROα) in 16 HNC patients [12]. The cytokines were analyzed with a multiplex assay and ELISA, pretreatment and post chemoradiotherapy, and one year post diagnosis. They increased significantly post treatment due to the response to therapy [12].

Citrin et al., in 2012, demonstrated that salivary IL-6, IL-8, EGF, IL-4, TNF-α, VEGF, and monocyte chemoattractant protein-1 (MCP-1) could predict for chemoradiotherapy toxicity or tumor control [13]. Salivary samples of 11 HNC patients were taken from low-dose and high-dose irradiation areas, pre-radiotherapy, and at three treatment fractions. Cytokine levels were elevated at the high dose area, in contrast with the low dose area. These biomarkers could be a tool for monitoring local changes in tissues during radiotherapy [13].

In 2022, Principe et al. confirmed that salivary IL-8 in HNC patients following radiotherapy could be a predictive biomarker of response to the treatment [14]. They analyzed salivary IL-6, IL-8, EGF, TNF-α, VEGF, IL-4, IL-10, and MCP-1 in 30 HNC patients before and after radiotherapy. They also compared these results with healthy cases. Salivary IL-8 increased statistically significantly in HNC patients post treatment, indicating good radiotherapy outcomes [14].

Alturfi et al., in 2025, explored the association of salivary EGF and the development of oral mucositis in HNC patients after radiotherapy [15]. Saliva samples were collected before radiotherapy, and two weeks and four weeks after treatment. Salivary EGF was measured with ELISA. Increased EGF expression was reported post treatment and correlated with reduced oral mucositis occurrence. An EGF concentration of 502.1 pg/ml marked a transition point for predicting mucositis following radiotherapy [15].

On the other hand, Epstein et al. in 2000 suggested that salivary EGF modified oral mucositis, induced by radiotherapy [16]. Salivary samples, unstimulated and stimulated, were gathered from 18 individuals, prior to and on a weekly basis during their course of radiation therapy. Assessment of oral mucositis was performed with the Oral Mucositis Assessment Scale (OMAS) and the National Cancer Institute Scale, evaluating the severity of redness, erythema, and ulcerations. Higher salivary EGF levels were statistically significantly associated with less severe erythema and ulceration. EGF was substantially reduced in the initial phase of treatment and was consistently reduced during treatment [16].

A study conducted by Dumbrigue et al. in 2000 showed that 13 HNC patients received radiotherapy and their EGF concentrations changed [17]. Saliva was collected before, during, and after radiotherapy. EGF was measured with ELISA, and the severity of oral mucositis was reported. Salivary EGF was decreased, and more severe mucositis was present, with statistically significant correlation [17].

Finally, Epstein et al. in 1997 reported the presence of EGF in 16 HNC patients during radiotherapy [18]. EGF was measured with ELISA, and radiotherapy-induced oral mucositis was evaluated with scores. Salivary EGF was decreased during the course of treatment, and an association was reported with reducing EGF and worsening ulcers in the oral cavity, and elevating total mucositis score and severity of oral mucositis [18].

Discussion

HNC sets a significant healthcare problem globally. Thus, there is a necessity to establish precise biomarkers for effective management. Several studies have reported increases in salivary cytokines in HNC patients than in individuals without cancer, but did not document altered expression of cytokines after radiotherapy [5,6].

IL-6 has plenty of biological activities. It acts as both an anti-inflammatory and a pro-inflammatory cytokine. It is responsible for the degradation of tissue via matrix metalloproteinase activation. It increases the migration of inflammatory cells and the fibroblast proliferation at the site of injury [19]. Salivary IL-6 has emerged as a potential diagnostic and therapeutic biomarker in HNC. Only one study showed a decrease in IL-6 levels after radiotherapy and chemotherapy, indicating treatment outcomes [10]. On the other hand, several studies showed an increase in salivary IL-6 post treatment. It was associated with worse radiation-induced mucositis in the oral cavity, serving as a predictive tool for preliminary risk evaluation of patients [11].

A concurrent increase in salivary IL-6 and IL-8, mainly by nuclear factor-kappa B (NF-κB), an important factor regulating the growth and evolution of HNC, indicated a common transcription pathway [20]. The increase of IL-6 and IL-8 is a marker of tumor response in the treatment outcome and normal tissue toxicity [13]. It could be a tool for the prediction of local effects of radiotherapy on normal tissues and the development of severe toxicity [13].

IL-8 is a crucial cytokine and is produced early in the inflammatory process. It can be active at the inflammation site for a long period of time [14]. IL-8 has a chemotactic effect on macrophages, promotes epithelial cell proliferation and migration, and stimulates the expression of metalloproteinases in leukocytes [21]. IL-8 plays an important role in local inflammatory progression during radiotherapy [13]. An increase in salivary IL-8 after radiotherapy could be a predictive biomarker for better treatment outcomes.

Furthermore, EGF is also an important cytokine. It controls cell proliferation and binding to its receptor, and modifies gene regulation [22]. Saliva contains EGF, which is important for the preservation of the epithelial barrier and stimulates wound healing in the oral cavity [15,16]. One study showed that the increase of salivary EGF in HNC patients after radiotherapy could probably lead to less severe mucositis in the oral cavity and decrease the occurrence [15]. On the other hand, several studies highlighted the decrease of salivary EGF post radiotherapy [16-18]. Lower EGF levels are related to more severe oral mucositis. EGF plays a protagonist role in the development of mucosal breakdown due to radiation [17]. After radiotherapy, the reduction of salivary flow and volume led to a decrease in the lubricating and antimicrobial effects of saliva, accompanied by a decrease in EGF. As a result, the oral mucositis was more severe [17,18].

Proteins, such as IL-6, IL-8, and EGF, participate in interconnected regulatory pathways. So, an analysis that allows simultaneous measurement of plenty of cytokines from one sample could be very helpful. Biopsies or invasive techniques delay tissue healing after the delivery of radiation [23]. A minimally invasive technique, such as salivary sampling, could serve as a baseline prognostic tool.

There are limitations to this review. A short number of studies reported the association of salivary cytokines pre- and post radiotherapy [24]. The cytokines were not measured using the same techniques. Additional studies in larger numbers of patients will be needed to validate findings and identify novel prognostic biomarkers.

## Conclusions

Radiotherapy is a common treatment choice for HNC, with or without chemotherapy. Despite the numerous studies describing diagnostic salivary biomarkers for HNC, there are few studies regarding which biomarker has the best prognostic value for the treatment response. This review suggests that salivary samples of cytokines could provide a useful, easy, accurate, and non-invasive monitoring tool for prognosis. Salivary IL-6, IL-8, and EGF could have a strong association with severe toxicity, severe oral mucositis, and radiotherapy outcomes. They could be a potential target in the future to minimize the adverse effects of treatment. However, more studies and further research are needed to verify the prognostic significance of salivary cytokines.
